# Parkinson’s disease and chronic kidney disease trends and disparities in the US population aged over 25 years (1999–2023): Insights from the CDC WONDER database

**DOI:** 10.1097/MD.0000000000050827

**Published:** 2026-09-18

**Authors:** Raymond Haward, Nikhil Duseja, Harkanwar Sandhu, Shankar Biswas, Jai Kumar Rajavoor Muniswamy, Mobeen Abid, Pooja Prasad, Newton Ashish Shah, Rutvik Jadvani, Iswariya Mani, Swathi Deepak Kulkarni

**Affiliations:** a Department of Internal Medicine, Trinity Health Oakland/Wayne State University, MI; b Department of Internal Medicine, Karachi Medical and Dental College, Karachi, Pakistan; c Department of Internal Medicine, Trinity Health Livonia/Wayne State University, MI; d Department of Internal Medicine, Ivano-Frankivsk National Medical University, Ivano-Frankivsk, Ukraine; e Department of Internal Medicine, Mayo Clinic, Jacksonville, FL; f Department of Public Health, Johns Hopkins Bloomberg School of Public Health, Baltimore, MD; g Department of Internal Medicine, Tribhuwan University Teaching Hospital, Kathmandu, Nepal; h Department of Internal Medicine, Government Medical College, Surat, India; i Department of Internal Medicine, UPMC Medical Educational Program, Pittsburg, Pennsylvania; j Department of Internal Medicine, HCA Healthcare/Tuft School of Medicine: Portsmouth Regional Hospital, New Hampshire, UK.

**Keywords:** adjusted mortality rate, CDC WONDER, chronic kidney disease, Parkinson’s

## Abstract

Parkinson’s disease (PD) is a progressive neurodegenerative disorder characterized by dopaminergic neuronal loss and α-synuclein aggregation, resulting in motor dysfunction and increasing mortality. Chronic kidney disease (CKD), affecting nearly 700 million people worldwide, is associated with systemic inflammation, vascular dysfunction, and excess mortality. Emerging evidence suggests a bidirectional relationship between CKD and PD, with renal impairment potentially accelerating neurodegeneration and worsening clinical outcomes. This study examined national mortality trends among individuals with coexisting PD and CKD in the United States from 1999 to 2023. This study followed the Strengthening the Reporting of Observational Studies in Epidemiology (STROBE) guidelines. Mortality data were obtained from the CDC WONDER database. Deaths listing both PD (International Classification of Diseases, Tenth Revision [ICD-10]: G20) and CKD (ICD-10: N18) were identified. Age-adjusted mortality rates (AAMRs) per 1,00,000 population were standardized to the 2000 US population and stratified by year, sex, region, and urban-rural status. Joinpoint regression estimated Annual percent change (APC), with *P* < .05 considered statistically significant. Between 1999 and 2023, 20,437 deaths were attributed to coexisting PD and CKD. National AAMR increased more than 4-fold, from 0.1 to 0.5 per 1,00,000 (APC = 5.23; 95% CI: 3.92–6.55). Mortality increased in females (0.1–0.3; APC = 5.15; 95% CI: 3.35–6.99) and males (0.2–0.8; APC = 4.79; 95% CI: 3.57–6.02). All census regions demonstrated significant increases, with the greatest rise in the West (APC = 5.92; 95% CI: 4.22–7.64). Mortality also increased in both urban (APC = 5.01) and rural (APC = 4.78) populations. Mortality among individuals with coexisting PD and CKD has risen substantially across demographic and geographic groups in the United States over the past 2 decades. These findings highlight the need for integrated neurology-nephrology care, early recognition of renal dysfunction, and targeted risk-factor management. Further research is warranted to clarify underlying mechanisms and develop strategies to reduce mortality in this high-risk population.

## 1. Introduction

Parkinson’s disease (PD) is a progressive neurodegenerative disorder characterized primarily by the loss of dopaminergic neurons in the substantia nigra pars compacta, resulting in dopamine deficiency within the nigrostriatal pathway leading to motor symptoms like bradykinesia, rigidity, and tremor.^[1]^ The degeneration occurs due to misfolding and aggregation of α-synuclein into Lewy bodies in these neurons, which disrupts synaptic signaling, impairs mitochondrial function, and activates neuronal apoptosis.^[2]^ Risk factors include aging, environment exposure to pesticides and heavy metals, chronic traumatic brain injury, and sedentary life that predisposes to alpha synuclein aggregation.^[3]^ Epidemiologically, PD is now the 14th leading cause of death in the United States, and age-adjusted mortality rates have risen significantly over the past 2 decades.^[4]^ Globally, an estimated 10.76 million people are living with PD, contributing to approximately 7.10 million/year/ year disability-adjusted life years (DALYs) lost annually.^[5]^ Notably, PD is the fastest-growing neurological disorder worldwide, with prevalence expected to double by 2040, largely driven by aging populations and environmental risk exposures.^[6]^

Chronic kidney disease (CKD) is defined as a persistent abnormality of kidney structure or function for ≥3 months, commonly measured by a reduced estimated glomerular filtration rate (eGFR < 60 mL/min/1.73 m^2^) or evidence of kidney damage such as albuminuria, structural abnormalities, or histologic changes.^[7]^ The 2 major global causes are diabetes mellitus and hypertension.^[8]^ Other causes include Glomerular Diseases, Polycystic Kidney Disease, Recurrent or Chronic Kidney Infections (Pyelonephritis) and Obstruction of Urinary Outflow (Post-renal CKD).^[9]^ In the United States and the world, CKD has emerged as a leading cause of death and approximately 697 million people worldwide are affected by it.^[10,11]^ Mortality is significantly higher when CKD coexists with diabetes, cardiovascular disease, or hypertension.^[12]^ Study by Yuan et al highlights the propagation of α-Syn pathology from kidney to the brain leading to understanding that CKD may facilitate or initiate neurodegeneration leading to PD.^[13]^

Given the significant mortality burden associated with both PD and CKD, this study evaluates mortality trends across these diseases to support public health planning and inform targeted strategies to reduce preventable deaths.

## 2. Materials and methods

### 2.1. Study setting and population

This study is a retrospective mortality analysis examining deaths associated with PD and CKD across the United States over a 25-year surveillance period from 1999 to 2023. Mortality data were obtained from the Centers for Disease Control and Prevention Wide-Ranging Online Data for Epidemiologic Research database (CDC WONDER), a publicly available and nationally authoritative repository that compiles death certificate records from all 50 states and the District of Columbia.^[14]^ Urbanization-classified data were available only through 2020 and were therefore analyzed over the restricted period from 1999 to 2020 accordingly. Causes of death were ascertained using the International Statistical Classification of Diseases and Related Health Problems, Tenth Revision. This analysis focused on International Classification of Diseases, Tenth Revision (ICD-10) code G20 for PD and ICD-10 code N18 for CKD, examining instances in which either condition was recorded as a multiple (contributing) cause of death.^[15]^ The dataset is provided by the US government under strict protocols that ensure the confidentiality and anonymity of individuals. The study was conducted in accordance with the Strengthening the Reporting of Observational Studies in Epidemiology (STROBE) guidelines for observational research.^[16]^

### 2.2. Data extraction

Given that PD and CKD frequently manifest as contributing, rather than primary, causes of death, a Multiple Cause of Death analysis was employed to capture the comprehensive mortality burden associated with each condition.^[17]^ This approach identifies all death certificates in which ICD-10 codes G20 or N18 were recorded in any cause of death field, thereby providing a more inclusive and representative estimate of PD and CKD-associated mortality across the study period than would be afforded by an underlying cause of death analysis alone. Data were extracted on key demographic and geographic variables, including annual mortality counts, sex-stratified mortality rates, regional geographic variation, and urbanization patterns associated with PD and CKD across all age groups from 1999 to 2023. Urbanization-classified data were available only through 2020 and were therefore analyzed over the restricted period from 1999 to 2020 accordingly. All demographic classifications were derived from self-reported information recorded on death certificates and are consistent with the methodology of prior studies utilizing the Centers for Disease Control and Prevention Wide-Ranging Online Data for Epidemiologic Research database. Sex was classified as male or female. Geographic regions, comprising the Midwest, Northeast, South, and West, were designated in accordance with United States Census Bureau definitions, as previously described by the Centers for Disease Control and Prevention and the National Center for Health Statistics and by Ingram and Franco.^[18]^ Urbanization was classified according to the National Center for Health Statistics Urban-Rural Classification Scheme, which stratifies counties into large metropolitan areas with a population of 1 million or more, medium and small metropolitan areas with populations ranging from 50,000 to 9,99,999, and rural areas with populations of fewer than 50,000 residents.^[18]^

### 2.3. Statistical analysis

To evaluate national mortality trends associated with PD and CKD in the United States, crude mortality rates and age-adjusted mortality rates per 1,00,000 population were calculated for the full study period spanning 1999 to 2023. Mortality rates were stratified by calendar year, sex, and geographic region across the entire study period, while urbanization-stratified analyses were restricted to the period from 1999 to 2020 in accordance with data availability constraints. All rate estimates were reported with corresponding 95% confidence intervals. Crude mortality rates were derived by dividing the total number of deaths in which PD or CKD was recorded as a cause of death by the corresponding United States population estimate for each calendar year. Age-adjusted mortality rates were standardized to the 2000 United States standard population to ensure comparability of estimates across time periods and demographic subgroups.^[19]^

Temporal trends in age-adjusted mortality rates were evaluated using Joinpoint regression analysis, performed using the Joinpoint Regression Program version 5.2.0.0 developed by the National Cancer Institute. This method applies log-linear regression models to identify statistically significant inflection points at which the direction or magnitude of a mortality trend undergoes a meaningful shift.^[20]^ Annual percent change (APC) values with corresponding 95% confidence intervals were calculated to quantify the direction and magnitude of change in age-adjusted mortality rates across each identified trend segment. APC estimates were considered statistically significant when the slope of the regression line differed significantly from zero, as determined by 2-tailed *t* tests at a significance threshold of *P* < .05.^[20]^ This analytic framework was selected for its capacity to account for evolving demographic compositions and shifting comorbidity burdens over the study period, both of which may independently influence observed mortality trends in PD and CKD.

### 2.4. Protocol approval and patient consent

This analysis was conducted using publicly available, fully deidentified mortality data obtained from the Centers for Disease Control and Prevention Wide-Ranging Online Data for Epidemiologic Research database. As no individually identifiable patient-level information was accessed or utilized at any stage of this study, the research was exempt from Institutional Review Board review and waiver of informed consent, in compliance with 45 CFR §46.104, the federal regulation governing exempt human subjects research.

## 3. Results

From 1999 to 2023, a total of 20,437 deaths were attributed to CKD and PD in the United States. The age-adjusted mortality rate (AAMR) increased significantly from 1999 to 2023 (0.1 to 0.5; Annual percentage change (APC) = 5.23; 95% CI: 3.92–6.55; *P* < .000001; Tables 1 and 2).

**Table 1:** Total deaths and sex-stratified mortality (male and female) among individuals with Parkinson’s disease (PD) and chronic kidney disease (CKD).

Year	US population	Total heart block deaths	Male deaths	Female deaths
1999	21,90,84,800	294	178	116
2000	22,11,68,531	367	252	115
2001	22,45,18,698	363	212	151
2002	22,70,62,163	390	264	126
2003	22,94,79,283	461	313	148
2004	23,21,53,496	490	314	176
2005	23,49,97,553	522	341	181
2006	23,78,63,203	509	342	167
2007	24,05,49,592	476	336	140
2008	24,31,86,582	419	288	131
2009	24,56,83,948	428	270	158
2010	24,75,18,325	427	276	151
2011	25,03,90,811	1092	696	396
2012	25,27,69,942	1189	754	435
2013	25,50,39,716	704	468	236
2014	25,77,89,101	762	509	253
2015	26,04,02,033	784	536	248
2016	26,21,52,444	883	590	293
2017	26,46,97,626	1034	693	341
2018	26,62,81,990	1147	763	384
2019	26,76,68,677	1256	828	428
2020	26,91,90,697	1614	1066	548
2021	27,13,27,075	1604	1034	570
2022	27,38,50,170	1608	1057	551
2023	27,54,16,414	1614	1071	543

CKD = chronic kidney disease, PD = Parkinson’s disease.

**Table 2:** Age-adjusted mortality rates (AAMRs) among the overall population and stratified by sex (male and female) for deaths associated with Parkinson’s disease (PD) and chronic kidney disease (CKD).

AAMR (95% CI)			
Year	Overall	Male	Female
1999	0.1 (0.1–0.1)	0.2 (0.2–0.3)	0.1 (0.1–0.1)
2000	0.2 (0.1–0.2)	0.3 (0.3–0.4)	0.1 (0.1–0.1)
2001	0.2 (0.2–0.2)	0.3 (0.2–0.3)	0.1 (0.1–0.1)
2002	0.2 (0.2–0.2)	0.3 (0.3–0.4)	0.1 (0.1–0.1)
2003	0.2 (0.2–0.2)	0.4 (0.3–0.4)	0.1 (0.1–0.1)
2004	0.2 (0.2–0.2)	0.4 (0.3–0.4)	0.1 (0.1–0.1)
2005	0.2 (0.2–0.2)	0.4 (0.4–0.4)	0.1 (0.1–0.1)
2006	0.2 (0.2–0.2)	0.4 (0.3–0.4)	0.1 (0.1–0.1)
2007	0.2 (0.2–0.2)	0.4 (0.3–0.4)	0.1 (0.1–0.1)
2008	0.2 (0.2–0.2)	0.3 (0.3–0.3)	0.1 (0.1–0.1)
2009	0.2 (0.2–0.2)	0.3 (0.2–0.3)	0.1 (0.1–0.1)
2010	0.2 (0.2–0.2)	0.3 (0.2–0.3)	0.1 (0.1–0.1)
2011	0.4 (0.4–0.4)	0.7 (0.6–0.7)	0.2 (0.2–0.3)
2012	0.5 (0.4–0.5)	0.7 (0.7–0.8)	0.3 (0.2–0.3)
2013	0.3 (0.2–0.3)	0.4 (0.4–0.5)	0.1 (0.1–0.2)
2014	0.3 (0.2–0.3)	0.5 (0.4–0.5)	0.1 (0.1–0.2)
2015	0.3 (0.3–0.3)	0.5 (0.4–0.5)	0.1 (0.1–0.2)
2016	0.3 (0.3–0.3)	0.5 (0.5–0.6)	0.2 (0.1–0.2)
2017	0.3 (0.3–0.4)	0.6 (0.5–0.6)	0.2 (0.2–0.2)
2018	0.4 (0.3–0.4)	0.6 (0.6–0.7)	0.2 (0.2–0.2)
2019	0.4 (0.4–0.4)	0.7 (0.6–0.7)	0.2 (0.2–0.2)
2020	0.5 (0.5–0.5)	0.8 (0.8–0.9)	0.3 (0.3–0.3)
2021	0.5 (0.5–0.5)	0.8 (0.8–0.9)	0.3 (0.3–0.3)
2022	0.5 (0.5–0.5)	0.8 (0.8–0.9)	0.3 (0.3–0.3)
2023	0.5 (0.5–0.5)	0.8 (0.7–0.8)	0.3 (0.3–0.3)

AAMR = age-adjusted mortality rates, CI = confidence interval, CKD = chronic kidney disease, PD = Parkinson’s disease.

**Figure 1.**
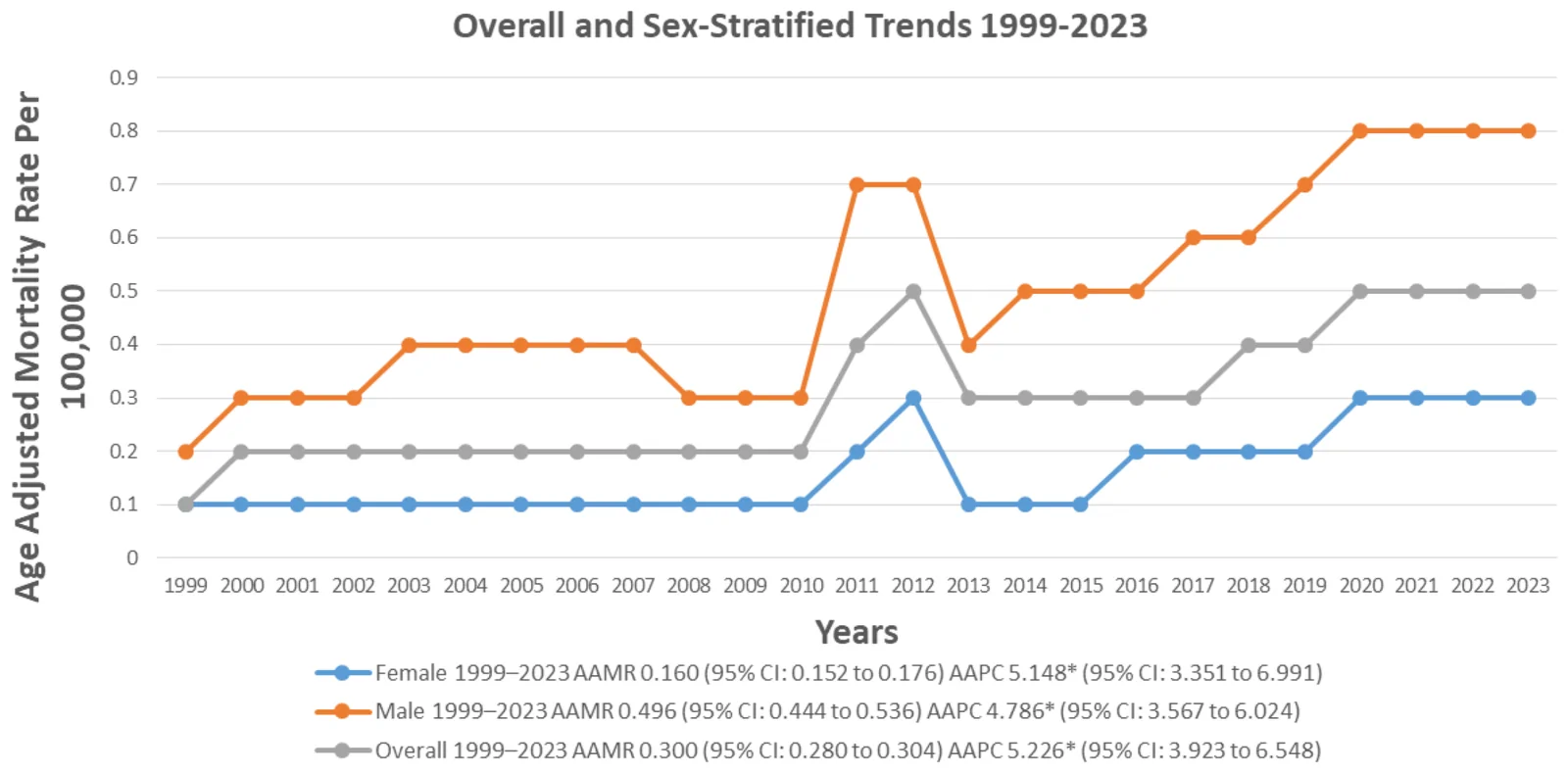
Overall and sex-stratified Parkinson’s disease and chronic kidney disease age-adjusted mortality rates per 1,00,000 adults in the United States, 1999 to 2023. AAMR = age-adjusted mortality rate, AAPC = age-adjusted annual percentage change, CI = confidence interval.

Sex-stratified analysis showed that among females, AAMR increased significantly from 1999 to 2023 (0.1–0.3; APC = 5.15; 95% CI: 3.35–6.99; *P* < .000001). Among males, AAMR significantly increased from 1999 to 2023 (0.2–0.8; APC = 4.79; 95% CI: 3.57–6.02; *P* < .000001). These results are depicted in Figure 1 and Tables 1 and 2.

**Figure 2.**
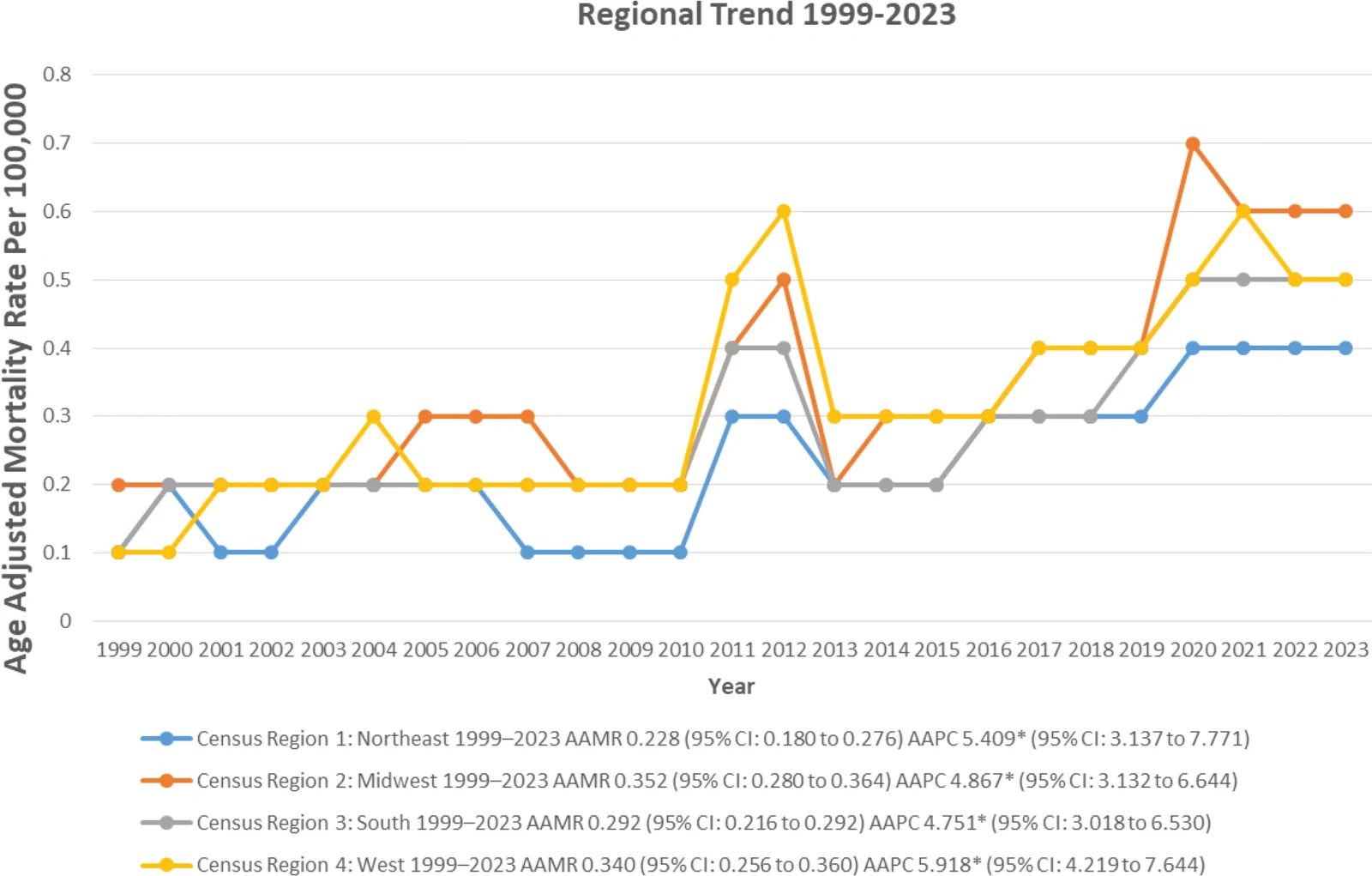
Region-stratified Parkinson’s disease and chronic kidney disease age-adjusted mortality rates per 1,00,000 adults in the United States, 1999 to 2023. AAMR = age-adjusted mortality rate, AAPC = age-adjusted annual percentage change, CI = confidence interval.

Regional trends showed that the Northeast region experienced a significant increase in AAMR from 1999 to 2023 (0.1–0.4; APC = 5.41; 95% CI: 3.14–7.77; *P* < .000001). The Midwest experienced a significant increase in AAMR from 1999 to 2023 (0.2 to 0.6; APC = 4.87; 95% CI: 3.13–6.64; *P* < .000001). The South experienced a significant increase in AAMR from 1999 to 2023 (0.1–0.5; APC = 4.75; 95% CI: 3.02–6.53; *P* < .000001). The West experienced a significant increase in AAMR from 1999 to 2023 (0.1–0.5; APC = 5.92; 95% CI: 4.22–7.64; *P* < .000001). These results are represented in Figure 2 and Tables 3 and 4.

**Table 3:** Overall deaths across US census geographic regions for Parkinson’s disease (PD) and chronic kidney disease (CKD).

Year	Northeast		Midwest			South		West
	Death	Population	Death	Population	Death	Population	Death	Population
1999	47	4,24,68,049	82	5,03,14,944	102	7,80,28,257	63	4,82,73,550
2000	72	4,26,92,596	110	5,05,87,672	125	7,89,41,529	60	4,89,46,734
2001	69	4,30,61,302	84	5,10,17,605	118	8,03,71,681	92	5,00,68,110
2002	68	4,33,46,662	114	5,13,14,670	133	8,15,08,351	75	5,08,92,480
2003	84	4,36,17,202	135	5,16,31,059	154	8,25,85,682	88	5,16,45,340
2004	86	4,38,19,373	125	5,19,46,501	159	8,39,32,308	120	5,24,55,314
2005	94	4,40,18,940	144	5,22,73,671	185	8,53,80,804	99	5,33,24,138
2006	84	4,42,29,025	147	5,26,19,995	165	8,68,06,861	113	5,42,07,322
2007	76	4,44,57,927	144	5,29,24,127	171	8,81,71,923	85	5,49,95,615
2008	68	4,47,41,455	126	5,31,89,240	145	8,94,46,027	80	5,58,09,860
2009	73	4,50,40,112	111	5,34,41,977	150	9,06,31,097	94	5,65,70,762
2010	75	4,52,54,463	118	5,36,37,169	141	9,15,08,374	93	5,71,18,319
2011	172	4,55,40,781	267	5,39,73,947	379	9,28,62,799	274	5,80,13,284
2012	158	4,58,28,012	301	5,41,98,561	400	9,40,11,879	330	5,87,31,490
2013	137	4,60,50,077	152	5,44,88,540	247	9,50,81,366	168	5,94,19,733
2014	125	4,63,04,430	210	5,47,50,576	233	9,63,95,376	194	6,03,38,719
2015	130	4,64,98,349	215	5,49,79,293	242	9,77,26,584	197	6,11,97,807
2016	147	4,65,00,752	221	5,50,77,712	299	9,87,80,134	216	6,17,93,846
2017	166	4,68,02,310	260	5,53,42,525	357	10,00,14,822	251	6,25,37,969
2018	201	4,65,45,043	283	5,55,21,758	389	10,10,70,908	274	6,31,44,281
2019	192	4,64,94,583	301	5,56,29,071	450	10,19,27,428	313	6,36,17,595
2020	225	4,64,33,303	457	5,56,99,847	566	10,29,97,343	366	6,40,60,204
2021	214	4,75,21,188	392	5,61,37,533	588	10,35,02,714	410	6,41,65,640
2022	229	4,76,14,213	424	5,63,62,716	553	10,52,03,667	402	6,46,69,574
2023	244	4,75,94,080	410	5,64,88,381	579	10,63,69,144	381	6,49,64,809

CKD = chronic kidney disease, PD = Parkinson’s disease.

**Table 4:** Age-adjusted mortality rates (AAMRs) across US census geographic regions for Parkinson’s disease (PD) and chronic kidney disease (CKD).

Year	AAMR (95% CI)			
	Northeast	Midwest	South	West
1999	0.1 (0.1–0.1)	0.2 (0.1–0.2)	0.1 (0.1–0.1)	0.1 (0.1–0.2)
2000	0.2 (0.1–0.2)	0.2 (0.2–0.3)	0.2 (0.1–0.2)	0.1 (0.1–0.2)
2001	0.1 (0.1–0.2)	0.2 (0.1–0.2)	0.2 (0.1–0.2)	0.2 (0.2–0.3)
2002	0.1 (0.1–0.2)	0.2 (0.2–0.2)	0.2 (0.1–0.2)	0.2 (0.1–0.2)
2003	0.2 (0.1–0.2)	0.2 (0.2–0.3)	0.2 (0.2–0.2)	0.2 (0.2–0.2)
2004	0.2 (0.1–0.2)	0.2 (0.2–0.3)	0.2 (0.2–0.2)	0.3 (0.2–0.3)
2005	0.2 (0.1–0.2)	0.3 (0.2–0.3)	0.2 (0.2–0.3)	0.2 (0.2–0.2)
2006	0.2 (0.1–0.2)	0.3 (0.2–0.3)	0.2 (0.2–0.2)	0.2 (0.2–0.3)
2007	0.1 (0.1–0.2)	0.3 (0.2–0.3)	0.2 (0.2–0.2)	0.2 (0.1–0.2)
2008	0.1 (0.1–0.2)	0.2 (0.2–0.3)	0.2 (0.1–0.2)	0.2 (0.1–0.2)
2009	0.1 (0.1–0.2)	0.2 (0.2–0.2)	0.2 (0.1–0.2)	0.2 (0.1–0.2)
2010	0.1 (0.1–0.2)	0.2 (0.2–0.2)	0.2 (0.1–0.2)	0.2 (0.1–0.2)
2011	0.3 (0.3–0.4)	0.4 (0.4–0.5)	0.4 (0.4–0.4)	0.5 (0.4–0.5)
2012	0.3 (0.2–0.3)	0.5 (0.4–0.5)	0.4 (0.4–0.5)	0.6 (0.5–0.6)
2013	0.2 (0.2–0.3)	0.2 (0.2–0.3)	0.2 (0.2–0.3)	0.3 (0.2–0.3)
2014	0.2 (0.2–0.3)	0.3 (0.3–0.4)	0.2 (0.2–0.3)	0.3 (0.3–0.4)
2015	0.2 (0.2–0.3)	0.3 (0.3–0.4)	0.2 (0.2–0.3)	0.3 (0.3–0.4)
2016	0.3 (0.2–0.3)	0.3 (0.3–0.4)	0.3 (0.2–0.3)	0.3 (0.3–0.4)
2017	0.3 (0.2–0.3)	0.4 (0.3–0.4)	0.3 (0.3–0.4)	0.4 (0.3–0.4)
2018	0.3 (0.3–0.4)	0.4 (0.4–0.5)	0.3 (0.3–0.4)	0.4 (0.3–0.4)
2019	0.3 (0.3–0.4)	0.4 (0.4–0.5)	0.4 (0.4–0.4)	0.4 (0.4–0.5)
2020	0.4 (0.3–0.4)	0.7 (0.6–0.7)	0.5 (0.4–0.5)	0.5 (0.5–0.6)
2021	0.4 (0.3–0.4)	0.6 (0.5–0.7)	0.5 (0.5–0.6)	0.6 (0.5–0.7)
2022	0.4 (0.3–0.4)	0.6 (0.6–0.7)	0.5 (0.4–0.5)	0.5 (0.5–0.6)
2023	0.4 (0.3–0.4)	0.6 (0.5–0.7)	0.5 (0.4–0.5)	0.5 (0.5–0.6)

AAMR = age-adjusted mortality rates, CI = confidence interval, CKD = chronic kidney disease, PD = Parkinson’s disease.

**Figure 3.**
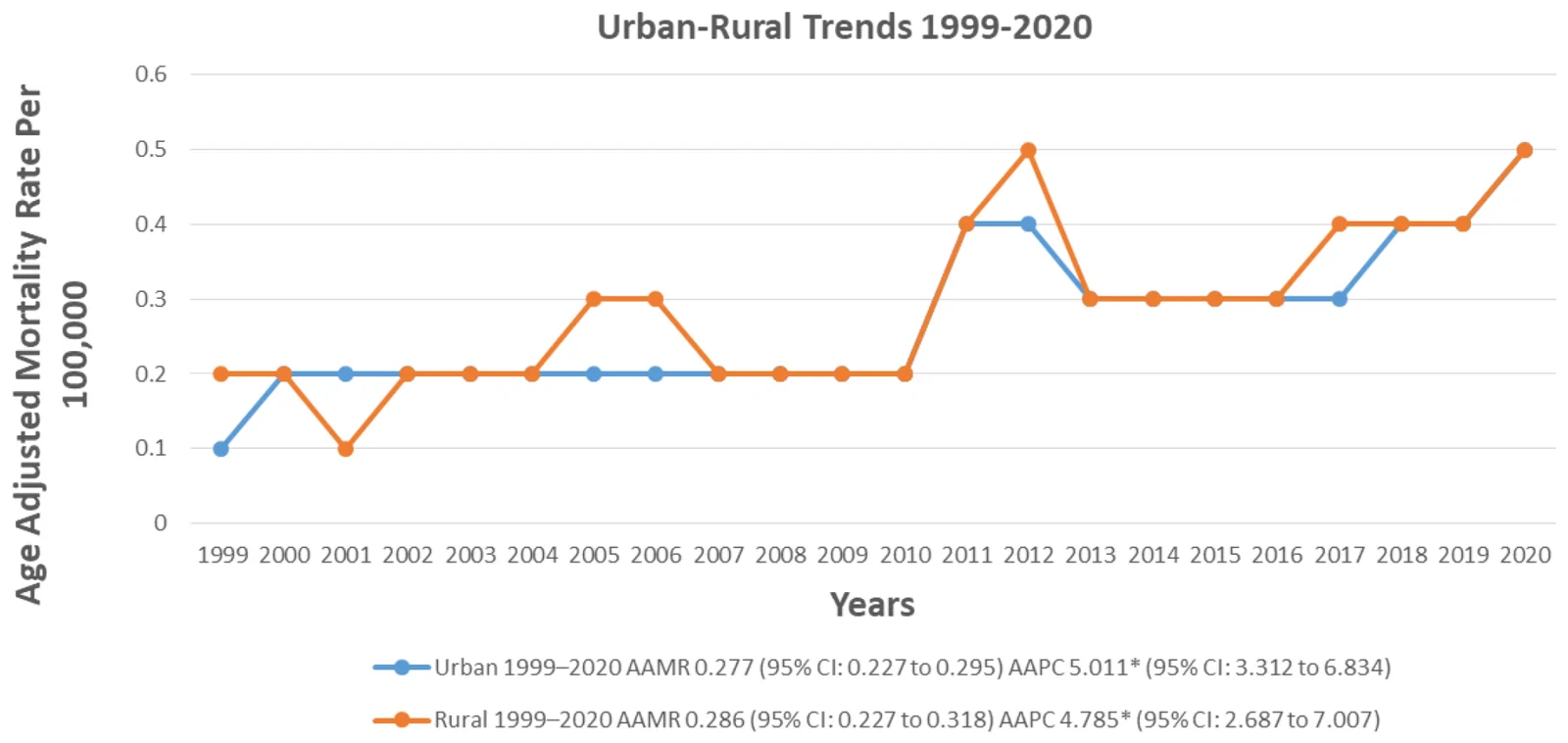
Urban and rural stratified Parkinson’s disease and chronic kidney disease age-adjusted mortality rates per 1,00,000 adults in the United States, 1999 to 2023. AAMR = age-adjusted mortality rate, AAPC = age-adjusted annual percentage change, CI = confidence interval.

Urbanization-specific trends showed that urban areas experienced a significant increase in AAMR from 1999 to 2020 (0.1–0.5; APC = 5.01; 95% CI: 3.31–6.83; *P* < .000001). Rural areas experienced a significant increase in AAMR from 1999 to 2020 (0.2–0.5; APC = 4.78; 95% CI: 2.69–7.01; *P* < .000001). These results are represented in Figure 3 and Tables 5 and 6.

**Table 5:** Overall deaths among rural and urban populations for Parkinson’s disease (PD) and chronic kidney disease (CKD).

Year	Urban population	Urban deaths	Rural population	Rural deaths
1999	18,37,31,694	220	3,53,53,106	74
2000	18,56,17,929	286	3,55,50,602	81
2001	18,87,98,253	303	3,57,20,445	60
2002	19,11,50,095	311	3,59,12,068	79
2003	19,33,54,753	371	3,61,24,530	90
2004	19,57,94,321	401	3,63,59,175	89
2005	19,83,89,174	400	3,66,08,379	122
2006	20,09,67,338	392	3,68,95,865	117
2007	20,34,48,956	376	3,71,00,636	100
2008	20,59,23,317	322	3,72,63,265	97
2009	20,83,11,115	333	3,73,72,833	95
2010	21,00,33,725	343	3,74,84,600	84
2011	21,28,04,208	893	3,75,86,603	199
2012	21,51,84,847	950	3,75,85,095	239
2013	21,74,07,626	573	3,76,32,090	131
2014	22,01,21,808	629	3,76,67,293	133
2015	22,26,97,459	628	3,77,04,574	156
2016	22,44,54,736	738	3,76,97,708	145
2017	22,69,93,727	829	3,77,03,899	205
2018	22,85,17,514	927	3,77,64,476	220
2019	22,98,85,901	1034	3,77,82,776	222
2020	23,13,80,941	1326	3,78,02,256	288

CKD = chronic kidney disease, PD = Parkinson’s disease.

**Table 6:** Age-adjusted mortality rates (AAMRs) among urban and rural populations with Parkinson’s disease (PD) and chronic kidney disease (CKD).

Year	AAMR (95% CI)	
	Urban	Rural
1999	0.1 (0.1–0.1)	0.2 (0.1–0.2)
2000	0.2 (0.1–0.2)	0.2 (0.1–0.2)
2001	0.2 (0.2–0.2)	0.1 (0.1–0.2)
2002	0.2 (0.1–0.2)	0.2 (0.1–0.2)
2003	0.2 (0.2–0.2)	0.2 (0.2–0.3)
2004	0.2 (0.2–0.2)	0.2 (0.2–0.3)
2005	0.2 (0.2–0.2)	0.3 (0.2–0.3)
2006	0.2 (0.2–0.2)	0.3 (0.2–0.3)
2007	0.2 (0.2–0.2)	0.2 (0.2–0.3)
2008	0.2 (0.1–0.2)	0.2 (0.2–0.3)
2009	0.2 (0.1–0.2)	0.2 (0.2–0.3)
2010	0.2 (0.1–0.2)	0.2 (0.1–0.2)
2011	0.4 (0.4–0.4)	0.4 (0.4–0.5)
2012	0.4 (0.4–0.5)	0.5 (0.4–0.6)
2013	0.3 (0.2–0.3)	0.3 (0.2–0.3)
2014	0.3 (0.2–0.3)	0.3 (0.2–0.3)
2015	0.3 (0.2–0.3)	0.3 (0.3–0.4)
2016	0.3 (0.3–0.3)	0.3 (0.2–0.3)
2017	0.3 (0.3–0.3)	0.4 (0.3–0.4)
2018	0.4 (0.3–0.4)	0.4 (0.4–0.5)
2019	0.4 (0.4–0.4)	0.4 (0.4–0.5)
2020	0.5 (0.5–0.5)	0.5 (0.5–0.6)

AAMR = age-adjusted mortality rates, CI = confidence interval, CKD = chronic kidney disease, PD = Parkinson’s disease.

## 4. Discussion

Significant upward trends in mortality were observed across all strata, including sex, geographic regions, and levels of urbanization. Evidence from a UK Biobank–based study reported that a reduced estimated glomerular filtration rate (eGFR) was significantly associated with an increased risk of PD.^[21]^ Additionally, Baik et al, in a cohort of 59,293 patients with newly diagnosed PD, found that those with eGFR < 60 mL/min/1.73 m^2^ had a higher risk of all-cause mortality (HR 1.24; 95% CI: 1.190–1.283) compared to individuals without CKD.^[22]^ In the same study, the presence of proteinuria further elevated mortality risk, with PD patients exhibiting a 54% higher risk of all-cause mortality compared to those without proteinuria (HR 1.543; 95% CI: 1.457–1.634).^[22]^ Collectively, these findings suggest that renal dysfunction represents a significant prognostic factor in PD, contributing to elevated mortality rather than being solely a renal complication.

CKD and PD share several underlying risk factors, including hypertension, diabetes, vascular disease, and oxidative stress, all of which contribute to both renal injury and neurodegeneration.^[23]^ Improvements in the management of these comorbid conditions – such as better glycemic control, cardiovascular therapies, and preventive care – have increased the overall survival of individuals with PD.^[24]^ As patients live longer, they are more likely to progress to advanced stages of the disease, where complications intrinsic to PD, rather than competing causes of mortality, become the dominant contributors to death.^[25]^ Thus, increased longevity may partially explain the rising mortality burden observed in recent years, particularly among PD patients with coexisting CKD.

Given the growing evidence that renal dysfunction is an independent predictor of mortality in PD, routine assessment of kidney function – including eGFR and markers of albuminuria or proteinuria – should be incorporated into standard clinical evaluation protocols for PD patients.^[22]^ Rather than viewing CKD as a separate renal comorbidity, it should be recognized as an indicator of systemic vascular and metabolic dysfunction that contributes to both disease progression and poorer survival outcomes.^[20]^ Strengthening care pathways that prioritize aggressive management of shared risk factors – such as hypertension, diabetes, cardiovascular disease, and metabolic syndrome – may help slow CKD progression while also reducing complications associated with advanced PD.^[26]^ Implementing multidisciplinary, comorbidity-focused clinical strategies and public health policies has the potential to improve long-term outcomes and mitigate the rising mortality burden observed among individuals living with PD complicated by CKD.

Patients with coexisting PD and CKD constitute a high-risk clinical subgroup, as both conditions independently contribute to frailty and functional decline. Together, they compound physiologic vulnerability, leading to reduced reserve and increased mortality.^[27]^ This population also has a disproportionately high risk of falls and fractures. PD causes gait instability, rigidity, and postural imbalance, whereas CKD contributes to muscle weakness, peripheral neuropathy, and orthostatic hypotension, resulting in a synergistic elevation in fall-related morbidity.^[28,29]^ The severity of fractures is further amplified by CKD-associated mineral bone disease, osteoporosis, and disturbances in calcium–phosphate homeostasis.^[30]^ Nutritional deterioration is another major concern; dysphagia and reduced caloric intake in PD, combined with protein-energy wasting in CKD, accelerate sarcopenia and cachexia, collectively worsening functional outcomes.^[31,32]^

Polypharmacy and medication toxicity further exacerbate frailty in this population. Antiparkinsonian medications, such as Amantadine, require renal dose adjustment, and impaired renal clearance increases susceptibility to adverse effects such as hallucinations, confusion, encephalopathy, and drug–drug interactions.^[33]^ Infection-related complications are also more common, as dysphagia in PD predisposes patients to aspiration pneumonia, while uremia-related immune dysfunction in CKD increases systemic vulnerability, cumulatively heightening infection-related mortality.^[34]^ Cardiovascular risk is elevated as well: CKD drives atherosclerosis, vascular calcification, and left ventricular hypertrophy, while the reduced cardiopulmonary reserve inherent to PD magnifies the risk of sudden cardiac death.^[24,35]^ Additionally, dialysis may worsen orthostatic hypotension, cognitive impairment, and malnutrition, and motor and cognitive impairments in PD may limit patients’ ability to access or tolerate dialysis effectively.^[26,36]^ CKD patients are often excluded from neurology trials, creating gaps in evidence-based dosing and treatment protocols for this population.^[37]^

### 4.1. Future research directions

Future investigations should further delineate the mechanisms and clinical consequences of coexisting PD and CKD. First, large longitudinal cohort studies are needed to disentangle cause-specific mortality patterns, distinguishing deaths attributable to neurodegeneration, cardiovascular disease, infections, renal failure, or frailty-related complications. Studies should also stratify by PD phenotype (e.g., tremor-dominant vs postural instability–gait disorder, early- vs late-onset, genetic vs idiopathic subtypes), as differential susceptibility to renal dysfunction may influence outcomes.^[38]^ Randomized clinical trials evaluating reno-protective therapies in PD, such as ACE inhibitors, SGLT2 inhibitors, renin–angiotensin blockade, phosphate binders, and mineral metabolism-modifying agents, may help identify interventions that mitigate mortality risk.^[39]^ Future work should also examine whether optimal renal management modifies the progression of motor and cognitive decline in PD.

Mechanistic studies are needed to characterize the kidney–brain axis, including uremic toxin–mediated neuroinflammation, endothelial dysfunction, mitochondrial injury, and altered dopamine metabolism.^[40]^ Integrative studies combining neuroimaging (DTI, fMRI, PET) with renal biomarkers (cystatin-C, NGAL, KIM-1) could clarify structural and metabolic pathways linking renal impairment to neurodegeneration. Additionally, research should address therapeutic challenges in pharmacokinetics and medication safety in PD + CKD populations, who are frequently excluded from clinical trials.^[41]^ Dose-adjustment frameworks for renally cleared agents and systematic evaluation of polypharmacy, drug toxicity, and dialysis-related symptom burden are needed to guide clinical practice. Population-level studies should evaluate social and geographic disparities, including differences by race, rurality, environmental exposure (e.g., pesticides, heavy metals, air pollution), healthcare access, and socioeconomic status.^[42,43]^ Understanding these gradients may clarify why mortality trends vary across demographic subgroups.

Finally, given the compounded disability burden, future research should focus on defining models of early palliative and supportive care integration for patients with dual diagnoses.^[44]^ The combined disease burden leads to complex symptoms such as pain, dysphagia, fatigue, dyspnea, malnutrition, mobility loss, and cognitive decline that necessitate interdisciplinary approaches involving neurology, nephrology, rehabilitation medicine, nutrition, psychiatry, and palliative care specialists. Studies evaluating timing, delivery models, quality-of-life outcomes, caregiver burden, and healthcare utilization would help establish evidence-based supportive care pathways.

### 4.2. Limitations

Although this study provides valuable insights into mortality trends among individuals with coexisting PD and CKD, several limitations should be acknowledged. First, the analysis is based on death certificate data obtained from CDC WONDER, which may be subject to misclassification or underreporting of underlying causes of death.^[45]^ Diagnostic coding accuracy may vary, particularly in elderly populations where multiple comorbidities complicate attribution, and CKD is frequently under-documented in mortality records.^[45]^ Second, the dataset lacks detailed clinical information, including disease severity, duration of PD, CKD stage, medication use, dialysis status, or presence of proteinuria. These clinical variables may influence both disease progression and mortality risk, limiting our ability to stratify outcomes by disease phenotype or treatment status. Similarly, the data do not capture comorbid conditions such as cardiovascular disease, diabetes, frailty, dysphagia, or aspiration history, all of which may mediate mortality in PD patients with renal impairment. Third, the study evaluates only fatal outcomes and does not include nonfatal clinical trajectories such as hospitalizations, falls, fractures, disability progression, or quality-of-life metrics. As a result, our findings describe mortality burden but do not characterize the broader morbidity profile of PD + CKD populations. Fourth, while place of death is recorded, the dataset does not provide information on disease management settings (e.g., home, outpatient care, skilled nursing facilities, dialysis centers), limiting our ability to analyze access to care, dialysis utilization, or differences in care delivery across regions. Fifth, mortality data do not distinguish whether deaths were directly attributable to PD, CKD progression, cardiovascular complications, infections, or neurodegenerative decline. Without linked person-level datasets (e.g., HCUP, Medicare, NEMSIS), we cannot determine whether rising mortality reflects an increase in disease incidence, worsening clinical outcomes, or improved documentation and attribution practices. Additionally, structural and socioeconomic factors such as rural–urban disparities, environmental exposure differences, insurance coverage, access to neurologists and nephrologists, and availability of preventive or palliative care services could contribute to regional mortality differences but were not evaluated in this study due to data limitations. Finally, Joinpoint regression assumes log-linear trends between inflection points, which may oversimplify nonlinear patterns influenced by demographic shifts, diagnostic advances, changes in ICD coding, or the impact of major healthcare disruptions such as the COVID-19 pandemic.^[46]^ Future research should incorporate linked, patient-level longitudinal data, stratified analyses by CKD stage and PD subtype, and advanced statistical approaches such as competing-risks models and age–period–cohort analyses to better understand causal pathways behind the observed mortality trends.^[47,48]^

## 5. Conclusion

This nationwide mortality analysis demonstrates a significant and sustained rise in deaths associated with coexisting PD and CKD in the United States from 1999 to 2023, with consistent increases across overall, sex, geographic regions, and urban–rural populations. These trends suggest that CKD is not merely a co-occurring condition but may serve as an independent prognostic factor that accelerates systemic vulnerability, exacerbates neurodegenerative progression, and worsens survival outcomes in PD. Shared risk factors, including vascular dysfunction, metabolic disease, aging, oxidative stress, and chronic inflammation, likely contribute to the synergistic mortality burden.

The clinical implications of these findings are substantial. Co-management models that integrate neurology and nephrology care, routine assessment of renal function in PD patients, optimization of cardiovascular and metabolic risk factors, and early implementation of reno-protective therapy may reduce disease progression and improve outcomes. Additionally, PD patients with CKD face unique therapeutic challenges, including altered pharmacokinetics, higher susceptibility to treatment toxicity, limited trial representation, dialysis-related complications, and escalating frailty. Addressing these gaps requires tailored clinical guidelines.

Future research should prioritize prospective cohort studies, mechanistic analyses of the kidney–brain axis, randomized trials of renal-protective interventions in PD, and stratified analyses across CKD stage, PD subtype, race, socioeconomic status, and environmental exposure. Adding palliative and supportive care earlier in the disease process may also improve quality of life and lower the cost of healthcare.

In summary, the rising mortality burden highlights an urgent need for targeted public health strategies and multidisciplinary clinical frameworks to address the complex and escalating challenges faced by individuals living with both PD and CKD.

## Author contributions

**Conceptualization:** Raymond Haward.

**Data curation:** Raymond Haward, Shankar Biswas.

**Formal analysis:** Iswariya Mani.

**Methodology:** Jai Kumar Rajavoor Muniswamy.

**Project administration:** Raymond Haward.

**Supervision:** Raymond Haward, Nikhil Duseja, Harkanwar Sandhu, Mobeen Abid.

**Validation:** Raymond Haward, Mobeen Abid.

**Writing – original draft:** Raymond Haward, Nikhil Duseja, Harkanwar Sandhu, Shankar Biswas, Jai Kumar Rajavoor Muniswamy, Mobeen Abid, Pooja Prasad, Newton Ashish Shah, Iswariya Mani.

**Writing – review & editing:** Newton Ashish Shah, Rutvik Jadvani, Swathi Deepak Kulkarni.

## References

[1] Zhou Z Yi L Wang Q Lim TM Tan EK. Role of dopamine in the pathophysiology of Parkinson’s disease. Transl Neurodegen. 2023;12:1. 10.1186/s40035-023-00378-6 PMC10506345 37718439

[2] Calabresi P Mechelli A Natale G Volpicelli-Daley L Di Lazzaro G Ghiglieri V. Alpha-synuclein in Parkinson’s disease and other synucleinopathies: from overt neurodegeneration back to early synaptic dysfunction. Cell Death Dis. 2023;14:1–16. 10.1038/s41419-023-05672-9 PMC9977911 36859484

[3] Huang M Bargues-Carot A Riaz Z. Impact of environmental risk factors on mitochondrial dysfunction, neuroinflammation, protein misfolding, and oxidative stress in the etiopathogenesis of Parkinson’s disease. IJMS. 2022;23:10808. 10.3390/ijms231810808 PMC9505762 36142718

[4] Martin J Osterman M. National vital statistics reports. Natl Vital Stat Rep. 2023;72:1. 36723449

[5] Peng S Liu P Wang X Li K. Global, regional and national burden of Parkinson’s disease in people over 55 years of age: a systematic analysis of the global burden of disease study, 1991-2021. BMC Neurol. 2025;25:178. 10.1186/s12883-025-04191-8 PMC12016353 40269818

[6] Dorsey ER Elbaz A Nichols E. Global, regional, and national burden of Parkinson’s disease, 1990–2016: a systematic analysis for the Global Burden of Disease Study 2016. Lancet Neurol. 2018;17:939–53. 10.1016/S1474-4422(18)30295-3 PMC6191528 30287051

[7] Chen A Sun Y Li W. Application of GFR estimation equations in elderly patients with measured GFR below 60 mL/min/1.73 m2. Aging Clin Exp Res. 2019;32:415–22. 10.1007/s40520-019-01218-2 31115878

[8] Ghaderian SB Beladi-Mousavi SS. The role of diabetes mellitus and hypertension in chronic kidney disease. J Renal Inj Prev. 2014;3:109–10. 10.12861/jrip.2014.31 PMC4301389 25610891

[9] Williams E Padmasayee P Sanjay B Harber M. Infections and the Kidney. Springer EBooks; 2022: 543–564

[10] Kovesdy CP. Epidemiology of chronic kidney disease: an update 2022. Kidney Int Suppl (2011). 2022;12:7–11. 10.1016/j.kisu.2021.11.003 PMC9073222 35529086

[11] Li M-J Liu H-Y Zhang Y-Q Li S-R Zhang J-H Li R. Global burden of chronic kidney disease and its attributable risk factors (1990-2021): an analysis based on the global burden of disease study. Front Endocrinol (Lausanne). 2025;105:1563246. 10.3389/fendo.2025.1563246 PMC12267038 40678313

[12] Nishizawa K Tanaka M Sato T. The health stage of cardiovascular-kidney-metabolic (CKM) syndrome is useful for predicting all-cause mortality in patients with type 2 diabetes: a cohort study in a period prior to the standard use of recent pharmacotherapy. J Diabetes Complications. 2025;39:109146. 10.1016/j.jdiacomp.2025.109146 40769112

[13] Yuan X Nie S Yang Y. Propagation of pathologic α-synuclein from kidney to brain may contribute to Parkinson’s disease. Nat Neurosci. 2025;28:577–88. 10.1038/s41593-024-01866-2 39849144

[14] Centers for Disease Control and Prevention. CDC WONDER. n.d. Available at: http://wonder.cdc.gov/. Accessed August 25, 2025.

[15] Al Armashi AR Al Zubaidi A Elantably D Wang J Alkrekshi A. Racial Disparities in mortality trends from acute myeloid leukemia: a population-based study in the United States between 2000 and 2019. Blood. 2022;140:1438–1438.

[16] Cuschieri S. The STROBE guidelines. Saudi J Anaesth. 2019;13:S31–4. 10.4103/sja.SJA_543_18 PMC6398292 30930717

[17] Minhas AMK Sperling LS Al-Kindi S Abramov D. Underlying and contributing causes of mortality from CDC WONDER—insights for researchers. Am Heart J Plus. 2025;50:100499. 10.1016/j.ahjo.2025.100499 PMC11782113 39895921

[18] Ingram DD Franco SJ. 2013 NCHS urban-rural classification scheme for counties. Vital Health Stat. 2014;2:1–73. 24776070

[19] Anderson RN Rosenberg HM. Age standardization of death rates: Implementation of the year 2000 standard. Natl Vital Stat Rep. 1998;47:1–16. 9796247

[20] Kim H-J Fay MP Feuer EJ Midthune DN. Permutation tests for joinpoint regression with applications to cancer rates. Stat Med. 2000;19:335–51. 10.1002/(sici)1097-0258(20000215)19:3<335::aid-sim336>3.0.co;2-z 10649300

[21] Peng H Wu L Chen Q. Association between kidney function and Parkinson’s disease risk: a prospective study from the UK Biobank. BMC Public Health. 2024;24:2225. 10.1186/s12889-024-19709-x PMC11328353 39148063

[22] Baik K Kang M Park YJ. Chronic kidney disease, proteinuria, and mortality risk in patients with Parkinson’s disease: a 12-year longitudinal study. Front Aging Neurosci. 2025;17:1631079–1631079. 10.3389/fnagi.2025.1631079 PMC12597998 41221266

[23] Zuo M Chang Le Neale N. The relationship between kidney health and neurodegenerative diseases. Brain. 2025;148:2616–30. 10.1093/brain/awaf113 PMC12316016 40120077

[24] Grosu L Grosu A Crisan D Zlibut A Perju‑Dumbrava L. Parkinson’s disease and cardiovascular involvement: edifying insights (Review). Biomed Rep. 2023;18:25. 10.3892/br.2023.1607 PMC9944619 36846617

[25] Macleod AD Taylor KSM Counsell CE. Mortality in Parkinson’s disease: a systematic review and meta-analysis. Mov Disord. 2014;29:1615–22. 10.1002/mds.25898 24821648

[26] Liabeuf S Pepin M Franssen CFM. Chronic kidney disease and neurological disorders: are uraemic toxins the missing piece of the puzzle? Nephrol Dial Transplant. 2021;37:ii33–44. 10.1093/ndt/gfab223 PMC8713157 34718753

[27] Walker SR Brar R Eng F. Frailty and physical function in chronic kidney disease: the CanFIT study. Can J Kidney Health Dis. 2015;2:32. 10.1186/s40697-015-0067-4 PMC4560862 26346754

[28] Lima DP de-Almeida SB Bonfadini JC. Falls in Parkinson’s disease: the impact of disease progression, treatment, and motor complications. Dement Neuropsychol. 2022;16:153–61. 10.1590/1980-5764-DN-2021-0019 PMC9173793 35720647

[29] Hsu S Bansal N Denburg M. Risk factors for hip and vertebral fractures in chronic kidney disease: the CRIC study. J Bone Miner Res. 2024;39:433–42. 10.1093/jbmr/zjae021 PMC11262146 38477777

[30] Hu L Napoletano A Provenzano M. Mineral bone disorders in kidney disease patients: the ever-current topic. Int J Mol Sci. 2022;23:12223–12223. 10.3390/ijms232012223 PMC9603742 36293076

[31] Kacprzyk KW Milewska M Zarnowska A Panczyk M Rokicka G Szostak-Wegierek D. Prevalence of malnutrition in patients with Parkinson’s Disease: a systematic review. Nutrients. 2022;14:5194. 10.3390/nu14235194 PMC9738273 36501224

[32] Koppe L Fouque D Kalantar‐Zadeh K. Kidney cachexia or protein‐energy wasting in chronic kidney disease: facts and numbers. J Cachexia Sarcopenia Muscle. 2019;10:479–84. 10.1002/jcsm.12421 PMC6596400 30977979

[33] deVries T Dentiste A Di Lea C Pichette V Jacobs D. Effects of renal impairment on the pharmacokinetics of once-daily amantadine extended-release tablets. CNS Drugs. 2019;33:783–9. 10.1007/s40263-019-00651-1 PMC6669187 31342404

[34] Patel B Legacy J Hegland KW Okun MS Herndon NE. A comprehensive review of the diagnosis and treatment of Parkinson’s disease dysphagia and aspiration. Expert Rev Gastroenterol Hepatol. 2020;14:411–24. 10.1080/17474124.2020.1769475 PMC10405619 32657208

[35] Zoccali C Mallamaci F Adamczak M. Cardiovascular complications in chronic kidney disease: a review from the European Renal and Cardiovascular Medicine Working Group of the European Renal Association. Cardiovasc Res. 2023;119:2017–32. 10.1093/cvr/cvad083 PMC10478756 37249051

[36] Murray AM. Cognitive impairment in the aging dialysis and chronic kidney disease populations: an occult burden. Adv Chronic Kidney Dis. 2008;15:123–32. 10.1053/j.ackd.2008.01.010 PMC2504691 18334236

[37] Charytan D Kuntz RE. The exclusion of patients with chronic kidney disease from clinical trials in coronary artery disease. Kidney Int. 2006;70:2021–30. 10.1038/sj.ki.5001934 PMC2950017 17051142

[38] Stebbins GT Goetz CG Burn DJ Jankovic J Khoo TK Tilley BC. How to identify tremor dominant and postural instability/gait difficulty groups with the movement disorder society unified Parkinson’s disease rating scale: comparison with the unified Parkinson’s disease rating scale. Mov Disord. 2013;28:668–70. 10.1002/mds.25383 23408503

[39] Heerspink HJL Stefánsson BV Correa-Rotter R; DAPA-CKD Trial Committees and Investigators. Dapagliflozin in patients with chronic kidney disease. N Engl J Med. 2020;383:1436–46. 10.1056/NEJMoa2024816 32970396

[40] Xie Z Tong S Chu X Feng T Geng M. Chronic kidney disease and cognitive impairment: the kidney-brain axis. Kidney Dis. 2022;8:275–85. 10.1159/000524475 PMC9386403 36157262

[41] Zoccali C Blankestijn PJ Bruchfeld A. The nephrology crystal ball: the medium-term future. Nephrol Dial Transplant. 2020;35:222–6. 10.1093/ndt/gfz199 31598700

[42] Santos-Lobato BL. Towards a methodological uniformization of environmental risk studies in Parkinson’s disease. NPJ Parkinson’s Dis. 2024;10:86. 10.1038/s41531-024-00709-y PMC11024193 38632283

[43] Pyatha S Kim H Lee D Kim K. Association between heavy metal exposure and Parkinson’s disease: a review of the mechanisms related to oxidative stress. Antioxidants (Basel). 2022;11:2467. 10.3390/antiox11122467 PMC9774122 36552676

[44] Barros-Ferreira M-I Reis-Pina P. Integrating palliative care in Parkinson’s Disease and related disorders: a systematic review of patient and caregiver outcomes. Am J Hosp Palliat Care. 2025:10499091251393691. 10.1177/10499091251393691 41199227

[45] Mieno MN Tanaka N Arai T. Accuracy of death certificates and assessment of factors for misclassification of underlying cause of death. J Epidemiol. 2016;26:191–8. 10.2188/jea.JE20150010 PMC4808686 26639750

[46] Abid H Memon S Das V Fatima K Mukhlis M Vazaym M. National trends and demographic disparities in mortality involving co-recorded Parkinson’s disease and dementia in the United States, 1999-2025: a CDC WONDER analysis. NeuroSci. 2026;7:66. 10.3390/neurosci7030066 PMC13304537 42347154

[47] Berry SD Ngo L Samelson EJ Kiel DP. Competing risk of death: an important consideration in studies of older adults. J Am Geriatr Soc. 2010;58:783–7. 10.1111/j.1532-5415.2010.02767.x PMC2873048 20345862

[48] Rosenberg PS Anderson WF. Age-period-cohort models in cancer surveillance research: ready for prime time? Cancer Epidemiol Biomarkers Prev. 2011;20:1263–8. 10.1158/1055-9965.EPI-11-0421 PMC3132831 21610223

